# miR-19 targets PTEN and mediates high mobility group protein B1(HMGB1)-induced proliferation and migration of human airway smooth muscle cells

**DOI:** 10.1371/journal.pone.0219081

**Published:** 2019-06-27

**Authors:** Changchun Hou, Yan Chen, Xiaolin Huang, Qinghua Huang, Mengze Li, Xiaoyu Tan

**Affiliations:** 1 Department of Respiratory Medicine, the second affiliated hospital of Guangxi Medical University, Nanning, China; 2 Department of Intensive Care Unit, the second affiliated hospital of Guangxi Medical University, Nanning, China; 3 Department of Respiratory and Critical Care Medicine, the first affiliated hospital of Guangxi Medical University, Nanning, China; University of South Alabama Mitchell Cancer Institute, UNITED STATES

## Abstract

**Background:**

The abnormal proliferation and migration of airway smooth muscle (ASM) cells contributes to airway remodeling during asthma. MiR-19a has been demonstrated to promote cell proliferation and angiogenesis of several cancer types by regulating the PTEN/PI3K/AKT pathway. Our previous study has shown that High-mobility group box protein 1 (HMGB1) is involved in the pathogenesis of airway remodeling using a mouse model of chronic asthma. However, the effects of HMGB1 on proliferation and migration of ASM cells and its underlying mechanisms remain unknown.

**Methods:**

Human ASM cells were obtained by primary explant techniques. MiR-19a expression was evaluated using qRT-PCR. Cell proliferation and migration were evaluated by the CCK-8 and the transwell migration assays, respectively. Transfection studies of ASM cells were performed to identify the underlying mechanisms.

**Results:**

HMGB1 stimulated ASM cell proliferation and migration in a dose-dependent manner. The expression levels of miR-19a and the PTEN and AKT signaling proteins were also modulated by HMGB1. Functional studies indicated that overexpression of miR-19a enhanced the proliferation and migration of ASM cells, whereas inhibition of miR-19a decreased the proliferation and migration of ASM cells. Western blot analysis demonstrated that miR-19a negatively regulated PTEN expression and positively regulated p-AKT expression. MiR-19 only regulates the proliferation of HASM cells induced by HMGB1, but not PDGF, EGF, TGF-β1. Furthermore, we demonstrated that miR-19 contributed to the promoting effects of HMGB1 on ASM cells by targeting PTEN 3’-UTR.

**Conclusion:**

Our results demonstrated that HMGB1 induced proliferation and migration of ASM cells via the miR-19a /PTEN/AKT axis and provided direct evidence on the role of HMGB1 in ASM cells proliferation in vitro. The present study further indicated that miR-19a may be explored as a potential novel therapeutic target to reverse proliferation and migration of ASM cells.

## Introduction

High mobility group box-1 protein (HMGB1) is a chromosomal protein, which functions as a nuclear factor, and is considered an important mediator in tissue repair, inflammation, tumorigenesis and innate and adaptive immunities when it is present in the extracellular region [1]. Extracellular HMGB1 can bind to specific receptors, advanced glycation end products (RAGE) and/or toll like receptors (TLR) in order to cause activation of various signaling pathways including the PI3K/AKT pathway [2]. HMGB1 has been implicated in several clinical diseases, such as rheumatic disease, sepsis and cancer [3]. In our previous study [4], we observed that the sputum and plasma HMGB1 levels were elevated in asthmatic subjects. More importantly, in a recent study we demonstrated that inhibition of HMGB1 activity decreased the levels of inflammatory mediators in the lung, whereas it could reverse airway remodeling in a allergen-induced murine model of chronic asthma [5]. In the present study, we demonstrated that inhibition of HMGB1 activity decreased airway smooth muscle thickness in mice. However, the effects of HMGB1 on the function of ASM cells and the associated mechanism remain unknown.

Asthma is a chronic airway inflammatory disease characterized by airway remodeling [6]. Airway remodeling may lead to irreversible or partially reversible airflow obstruction and decreased lung function [7]. The abnormal airway smooth muscle (ASM) mass is one of the hallmarks of airway remodeling. The increased proliferation and migration of ASM cells contribute to the increase in the airway smooth muscle mass [8]. Therefore, it is very important and essential to further elucidate the mechanisms underlying the proliferation and migration of ASM cells.

MicroRNAs (miRNAs) is a large family of small noncoding RNAs that negatively regulate target messenger RNA (mRNA) by interacting with complementary sites in the 3′-untranslated region (UTR) of mRNA molecules [9]. Various studies have demonstrated that miRNAs play a vital role in allergic airway inflammation [10]. Notably, miR-19a is upregulated in T cells from asthmatic subjects, and it promotes TH2 cytokine production and airway inflammatory signaling by direct targeting of the signaling inhibitors PTEN(phosphatase and tensin homolog deleted on chromosome 10) [11]. PTEN has been widely identified as the key gene involved in the initiation, proliferation and metastasis of tumor cells [12]. In addition, miR-21 modulates human airway smooth muscle cell proliferation and migration in asthma by targeting PTEN[13]. Moreover, PTEN can exert its role as a suppressor by negatively regulating the PI3K(phosphatidylinositol(PI) 3-kinase)/Akt signaling pathway [14]. The PI3K/Akt signaling pathway has been shown to be involved in the proliferation, hypertrophy and migration of ASM cells [15]. Based on the aforementioned data, we hypothesized that HMGB1 exerts direct effects on the proliferation and migration of ASM cells *in vitro* by modulating the miR-19a/PTEN/AKT axis. Therefore, the present study aimed to confirm this hypothesis.

## Materials and methods

### Primary human airway smooth muscle (HASM) cell isolation and culture

In the present study, primary HSAM cells were obtained from the main bronchi of human non-tumor lung tissues from four patients (age range, 49–72, 2 males, and 2 females) undergoing lobectomy at the second Affiliated Hospital of the Guangxi Medical University (China). A protocol was approved by the Ethics Committee of the Guangxi Medical University(number:2016-gxmu-16). All patients who participated in the present study provided written informed consent. The trachea and main bronchi were dissected by removing the excess connective tissue and were washed in Phosphate buffered saline (PBS) solution with antibiotics (100 U/ml penicillin G and streptomycin). Subsequently, the epithelia were removed by slight stripping of the luminal surface and the tissues that were cut into small pieces. The samples were incubated in DMEM with 0.1% collagenase solution at 37°C for 4 h. The cell suspension was placed in a culture flask with complete DMEM/F12 and 10% FBS. The flasks were cultured at 37°C in a humidified incubator. Cultured HASM cells were identified by the expression of α-smooth muscle actin proteins. HASM cells that were grown between passage 4 and 6 were used for all experiments.

### Treatment of HASM cells

HASM cells were cultured in DMEM/F12 with 10% FBS and antibiotics (100 U/ml penicillin G and streptomycin), and were placed in a humidified incubator with 5% CO_2_ at 37°C. HASM cells were starved in serum-free DMEM/F12 medium for 24 h prior to treatment. The cell monolayer was allowed to grow at confluent levels and the cells were trypsinized and plated in 6-well plates. The cells were stimulated with HMGB1(Sigma, USA), PDGF, EGF and TGF -β1 at different concentrations for the indicated time periods (0, 12, 24 and 48 h). Subsequently, primary HASM cells were incubated with HMGB1 in the absence and/or presence of 25 μM of LY294002 (an inhibitor of the PI3K/AKT signaling pathway, Sigma, St Louis, MO, USA).

### Transfection of miR-19a mimics and inhibitor

HASM cells were transferred at a density of 2x10^5^ cells in 6-well plates and incubated for 24 h under the aforementioned conditions. The cells were transfected with 100 nM inhibitor negative control, 50 nM negative control mimics, 50 nM miR-19a mimics or 100 nM miR-19a inhibitor (RIBOBIO, Guangzhou, China) using lipofectamine 2000 reagent (Invitrogen, Carlsbad, USA) following the manufacturer’s protocol. Following 48 h of incubation, the expression levels of miR-19a were detected by Real-Time PCR.

### RNA extraction and real-time PCR

Total cellular RNA was isolated from HASM cells using Trizol reagent (Invitrogen, Carlsbad, CA, USA). The RNA quality was confirmed by calculating the OD260/280 ratio and the miR-19a expression was assessed using Bulge-Loop miRNA qRT-PCR Starter Kit based on stem-loop real-time PCR following the manufacturer's instructions (RIBOBIO,Guangzhou, China). Real-time PCR was performed on the ABI 7900 (Applied Biosystems, USA) using SYBR Green (RIBOBIO,China). The sequence for the stem-loop RT-PCR primer of miR-19a (5’-3’) was the following: GTCGTATCCAGTGCAGGGCCTGAGGTATTCGCACTGGATACGACTCAGTT. The nucleotide primers used for real-time PCR were as follows (5'-3'): miR-19a-F, CCTCTGTTAGTTTTGCATAGTTGC; U6-F,GCGCGTCGTGAAGCGTTC; Universal reverse primer GTGCAGGGTCCGAGGT (5’-3’). Each reaction was performed in triplicate. The U6 small nucleolar RNA was used to normalize miRNA measurements. Relative quantification of miR-19a expression was normalized to U6 and calculated using the ΔΔCT method.

### Cell proliferation assay

The Cell Counting Kit-8 (CCK-8) assay was used to evaluate HASM cell proliferation. Briefly, HASM cells were plated at a density of 4x10^3^cells/well in 96-well plates and serum-starved for 24 h. Subsequently, the cells were incubated with 10 μl of the CCK-8 solution for 2 h. The absorbance values of the samples were determined at 450 nm (A450) with a microplate reader. A total of 6 replicates were used for each group.

### Cell migration assay

The transwell method (Costar, Corning, NY, USA) was performed to evaluate HASM cell migration. Briefly, the transwell migration assay was conducted following cell trypsinization and resuspension (6*10^4^ cells/ml) in serum free growth medium. The cells were treated with miR-19a mimics or miR-19a inhibitor and added to the upper chamber. Furthermore, 500 μl of medium containing 10% FBS (chemoattractants) was added to the lower chamber and the cells were allowed to migrate for 24 h at 37°C. The non-migrated cells on the upper chamber were removed, whereas the migrated cells were fixed with 4% polyoxymethylene and stained with 0.1% crystal violet (Biyuntian, Shanghai, China). The number of migrated HASM cells was counted in five random fields.

### Western blotting assay

The procedure of Western blot analysis was performed as previously described [5]. Total protein content was isolated from HASM cells using a protein extraction lysis buffer (Biyuntian, China) in the presence of a protease inhibitor (PMSF, Biyuntian, China). The protein concentration of the cell lysates was quantified using a BCA protein assay kit (Biyuntian, China). An equal amount of protein derived from the different groups was separated on a 10% SDS gel. Following separation, the proteins were transferred to a nitrocellulose membrane (PALL, USA) and the membranes were incubated at room temperature in 5% non-fat milk in TBST. Subsequently, the membranes were incubated with rabbit polyclonal Abs against GAPDH and a-SMA (Immunoway, USA) and rabbit monoclonal Abs against AKT and phosphorylated AKT (Cell Signaling Biotechnology). The following morning, the membranes were incubated with a secondary antibody (goat anti-rabbit IgG H&L IRDye 800CW, Licor, USA) at room temperature for 1 h. The membranes were quantified by densitometry using the Odyssey infrared image system (Licor, USA). GAPDH was used as the control for the normalization of the relative protein expression levels.

### Dual-luciferase reporter assay

The wild-type (WT) or mutant (MUT) PTEN 3′-UTR dual luciferase reporter gene plasmids containing the predicted binding site of miR-19a were purchased from RIBOBIO (Guangzhou, China). HASM cells were seeded in 24-well plates and cultured for 24 h. PTEN-3′UTR-WT or MUT plasmids were transfected into HASM cells using lipofectamine 2000 reagent (Invitrogen). Following transfection, HMGB1 (2,000 ng/ml) was added to each well for 48 h. The *Renilla* luciferase reporter vector was used as the internal control. Luciferase activity was evaluated with the Dual-luciferase Reporter Assay Kit (Promega Corporation, Madison, USA) according to the manufacturer’s instructions. The experiments were performed in triplicate.

### Statistical analysis

All experimental data were expressed as the mean ± SEM. Statistical analyses were performed using the SPSS 16.0 software. A Student 2-tailed unpaired *t* test was selected to compare between two different groups.The comparisons between the different groups were analyzed using one-way ANOVA followed by the least significant difference (LSD) multiple comparison test. P values lower than 0.05 (P<0.05) were considered statistically significant.

## Results

### Effects of HMGB1 on proliferation and migration of HASM cells

**Fig 1:**
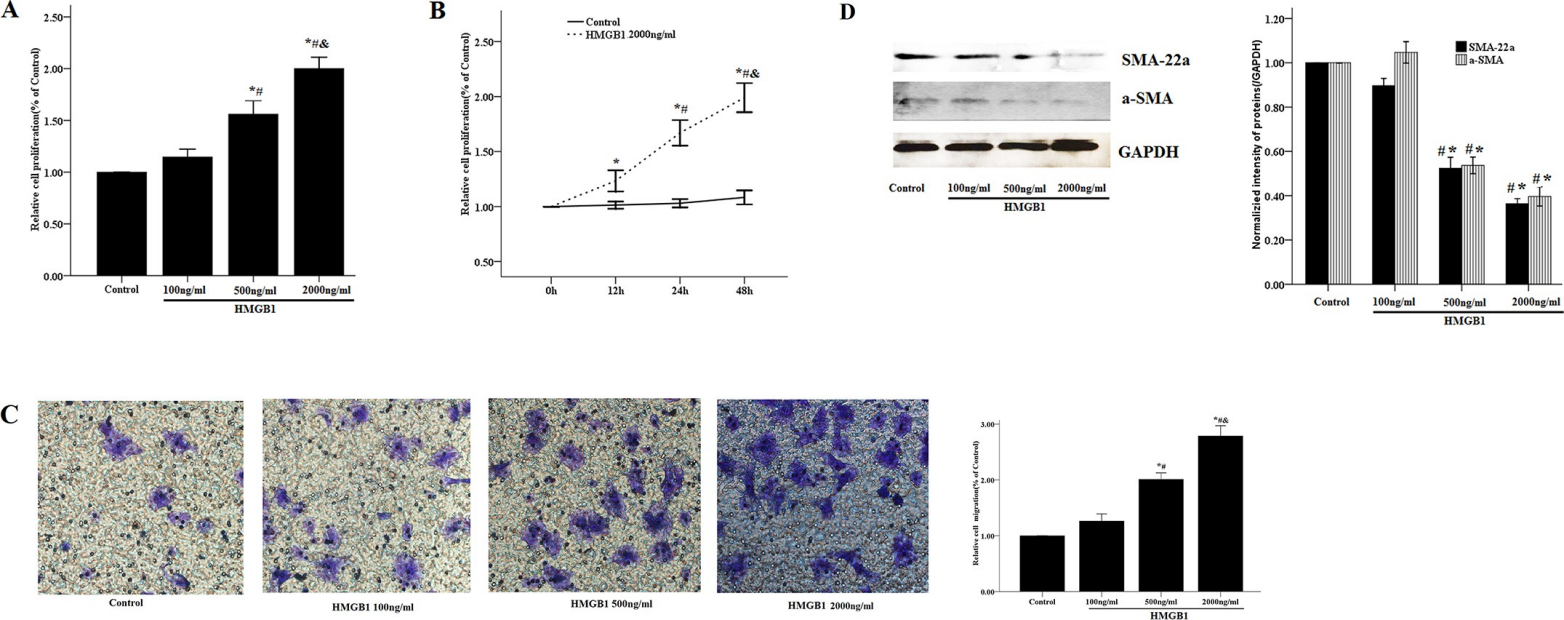
Effects of HMGB1 on proliferation and migration of HASM cells. (A) The CCK-8 assay was used to evaluate HASM cell proliferation. The primary HASM cells were treated with the indicated concentrations of HMGB1 (100–2,000 ng/ml) for 48 h. *P < 0.05 vs. control cells; #P < 0.05 vs. the cells treated with HMGB1 100 ng/ml; &P < 0.05 vs. the cells treated with HMGB1 500 ng/ml. (B) HASM cells were treated with HMGB1 2,000 ng/ml for 12, 24 and 48 h. *P < 0.05 vs. unstimulated cells at 0 h; #:P < 0.05 vs. the cells treated with HMGB1 for 12 h; &:P < 0.05 vs. the cells treated with HMGB1 for 24 h. (C)The transwell method was performed to evaluate HASM cell migration. (D)The SMA and SM-22a protein levels were detected by western blotting. HASM cells were treated with the indicated concentrations of HMGB1 (100–2,000 ng/ml) for 48 h. *P < 0.05 vs. control cells; #P < 0.05 vs. cells treated with HMGB1 100 ng/ml; GAPDH was selected as an internal control. The data were expressed as mean±SEM from four independent experiments.

It is widely accepted that HMGB1 promotes proliferation and migration (metastasis) of several tumor types, including breast, colon and skin (melanoma) [16]. In addition, HMGB1 can also enhance proliferation of pulmonary arterial smooth muscle cells and primary human arterial endothelial cells [17]. However, the role of HMGB1 on proliferation and migration of HASM cells *in vitro* has not been determined. In the present study, we found that HMGB1 significantly induced proliferation of HASM cells in a time- and dose-dependent manner compared with that of the vehicle control group (P<0.05) (Fig 1A and 1B). Similarly, HMGB1 could stimulate the migration of HASM cells in a concentration-dependent manner (Fig 1C). A total of 2,000 ng/ml of HMGB1 led to an almost 172% increase in cell migration compared with that of the control group (Fig 1C). Following treatment with several mitogenic stimuli, ASM cells exhibited reversible switching from the contractile phenotype to the proliferative phenotype [18]. ASM cells were characterized by high proliferative rates and low expression levels of contractile proteins, such as smooth muscle myosin heavy chain (SMMHC, SM-22a) and smooth muscle a-actin (a-SMA). Therefore, we sought to examine the effects of HMGB1 on the expression levels of SM-22a and a-SMA in HASM cells. As shown in Fig 1D, SM-22a and a-SMA protein expression of HASM cells were significantly decreased following HMGB1 stimulation.

### HMGB1 induces up-regulation of miR-19a

**Fig 2:**
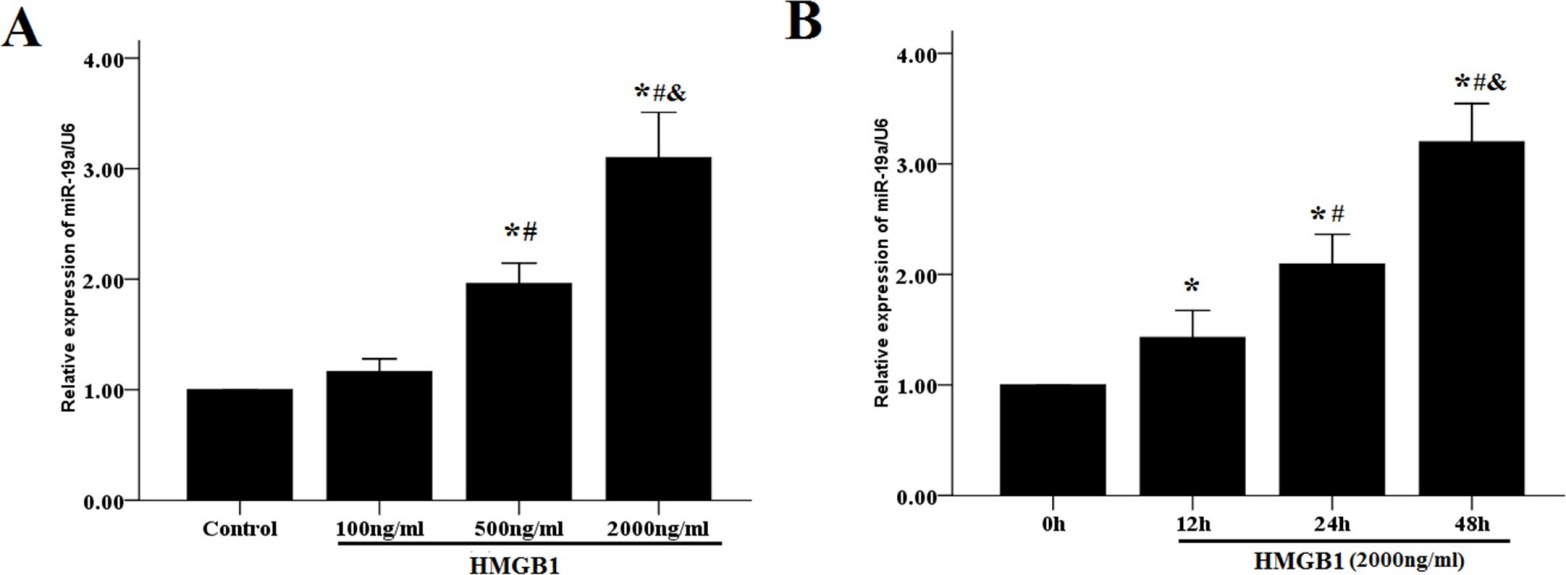
HMGB1 induces the expression of miR-19. Primary HASM cells were treated with indicated concentrations of HMGB1 (100–2,000 ng/ml) for 48 h (A) and HMGB1 2,000 ng/ml for 12, 24 and 48 h (B). U6 snRNA was used as an internal control. The data were expressed as mean±SEM from four independent experiments. (A)*P<0.05 vs. control cells; #P < 0.05 vs. the cells treated with HMGB1 100 ng/ml; & P<0.05 vs. the cells treated with HMGB1 500 ng/ml.(B)*: P < 0.05 vs. unstimulated cells at 0 h; #:P < 0.05 vs. the cells treated with HMGB1 for 12 h; &:P < 0.05 vs. the cells treated with HMGB1 for 24 h.

Overexpression of miR-19a is a key oncogenic event noted in various cancer types [19]. In the present study, we examined the alteration in the expression levels of miR-19a in HAMS cells following HMGB1 treatment. Real-time PCR analysis revealed that HMGB1 induced a significant increase in the expression levels of miR-19a in HASM cells in a dose-dependent manner (Fig 2A). In addition, HMGB1 (2,000 ng/ml) induced a marked upregulation of miR-19a in HASM cells in a time-dependent manner (Fig 2B). These data (S1 and S2 Figs) demonstrated that treatment with HMGB1 resulted in increased proliferation and migration of HASM cells that were concomitant with the up-regulation of miR-19a.It is well-known that some growth factors like PDGF, EGF and TGF-β1 could induce functional abnormalities of airway smooth muscle, so we also explored the effects of these growth factors on the miR-19a expression of HASM cells. As shown in S2 Fig, noly HMGB1 induces the expression of miR-19a in in HASM cells.

### HMGB1 activated the PTEN/AKT pathway in HASM cells

**Fig 3:**
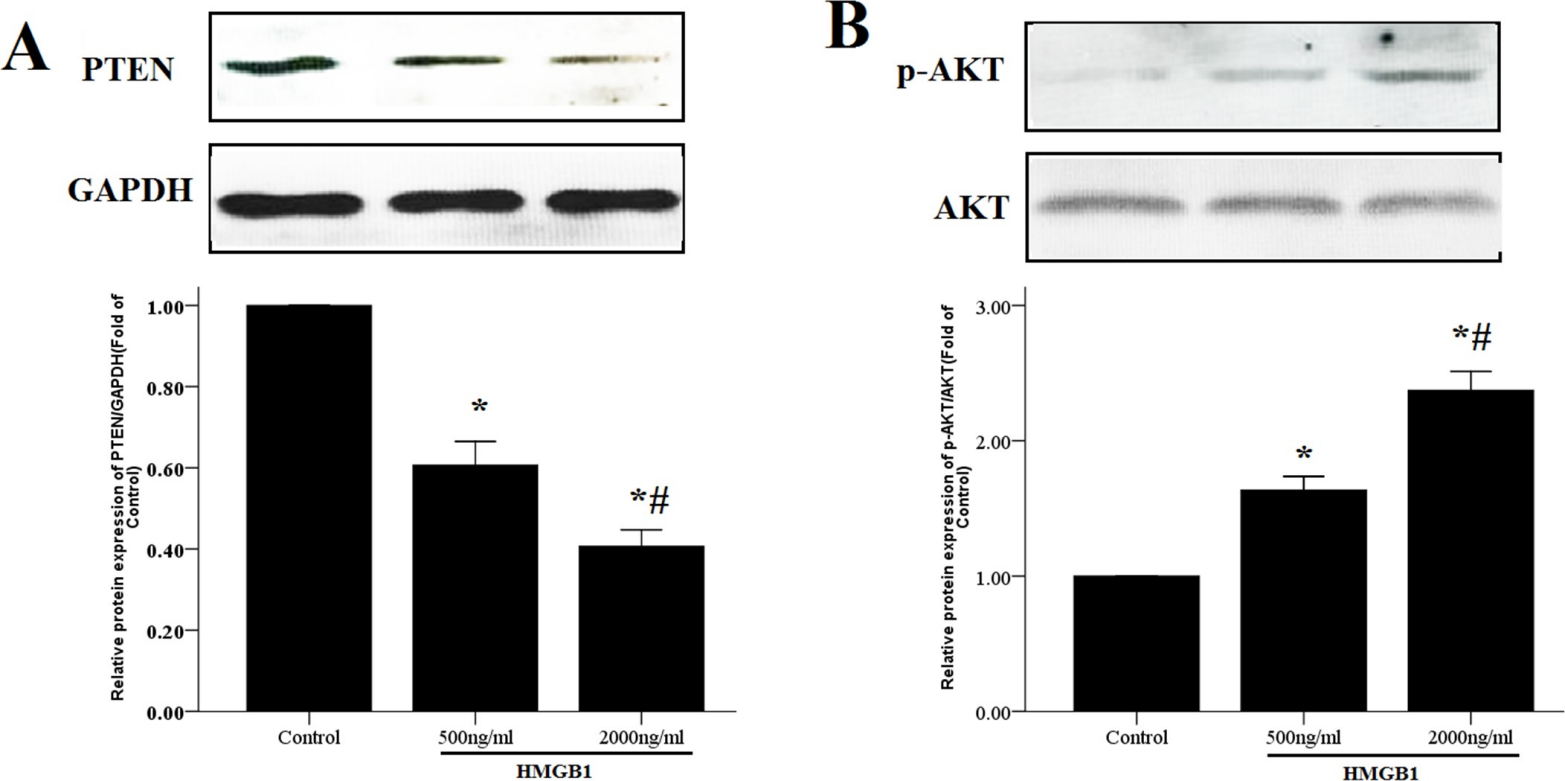
HMGB1 modulates PTEN/AKT axis in HASM cells. HASM cells were treated with HMGB1 (500 and 2,000 ng/ml) for 48 h and the protein levels of PTEN (A) and p-AKT (B) were determined by WB. GAPDH and t-AKT were used as the internal controls for PTEN and p-AKT, respectively. The data were expressed as mean±SEM from four independent experiments. *P<0.05 vs. control cells; #P < 0.05 vs. the cells treated with HMGB1 500 ng/ml.

PTEN has been identified as the important target gene of miR-19a in several types of cancer cells including ASM cells [12,13]. PTEN was reported as a suppressor of the PI3K/AKT pathway [14]. Therefore, we investigated the expression levels of PTEN and AKT in HASM cells in response to HMGB1 treatment. As shown in Fig 3A, we observed that the protein levels of PTEN were decreased by 40–60% following treatment of HMGB1 in HASM cells compared with those noted in the control group. In contrast to these observations, AKT phosphorylation was markedly increased following stimulation with HMGB1 (Fig 3B).

### miR-19a regulates HASM cell proliferation, migration, and expression of contractile marker proteins

**Fig 4:**
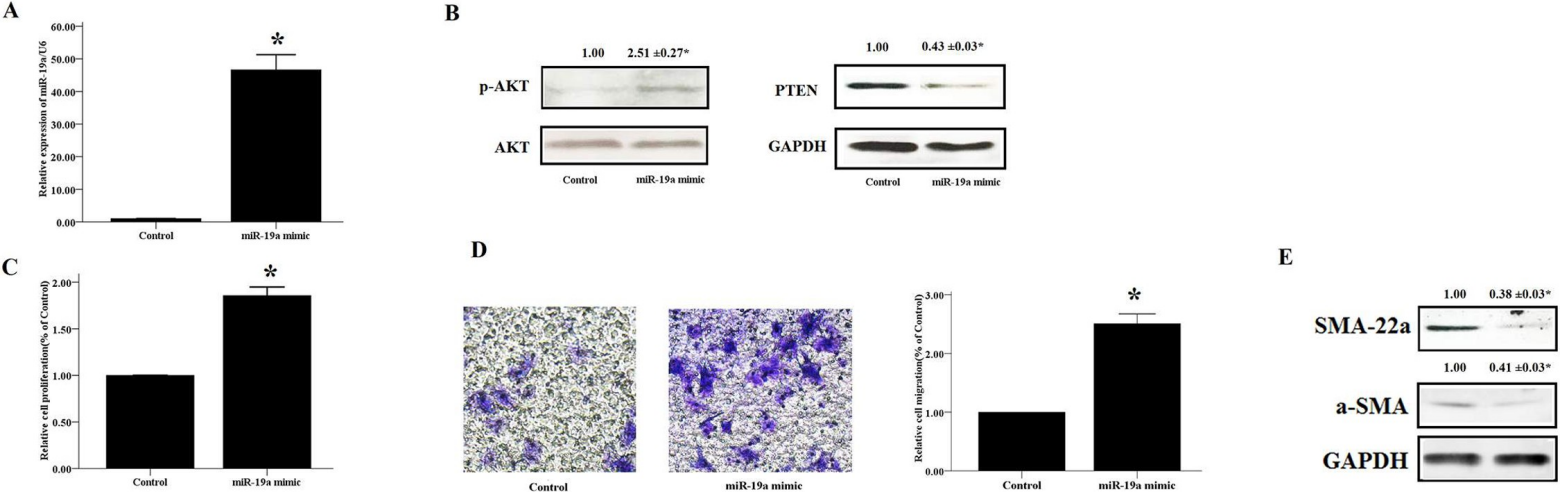
MiR-19a mimics accelerated the proliferation and migration of HASM cells. (A, B, E) HASM cells were transfected with 50 nM of miR-19a mimics and/or negative vector control and cultured for 48 h. The transfection efficiency was measured by Real-time PCR (A) and the protein levels of PTEN, p-AKT (B), SMA and a-SMA (E) were measured by western blotting. U6 snRNA was selected as an internal control. (C) The CCK-8 assay was used to evaluate HASM cell proliferation. (D) The transwell method was performed to evaluate HASM cell migration. The data were expressed as mean±SEM from four independent experiments. * P<0.05 vs. vector control cells.

We further investigated the potential role of miR-19a in regulating the proliferation, migration and expression of contractile marker proteins in HASM cells. MiR-19a mimics were transfected into HASM cells and the cells were incubated for 48 h. The levels of miR-19a were increased higher than 50-fold in HASM cells transfected with miR-19a mimics compared with those transfected with negative control miRNA mimics (Fig 4A). The increase noted in the miR-19a expression levels was accompanied with an increase in the proliferation and migration of the HASM cells that were treated with miR-19a mimics. In parallel, a marked induction of AKT phosphorylation was noted in these cells. In contrast to AKT, overexpression of miR-19a decreased the protein levels of PTEN, SM-22a and SMA proteins. These results indicated that miR-19a promoted cell proliferation and migration and regulated the PTEN/AKT pathway in the HASM cells.

### The miR-19a inhibitor suppressed the proliferation and migration of HASM cells and activated the PTEN/AKT pathway

**Fig 5:**
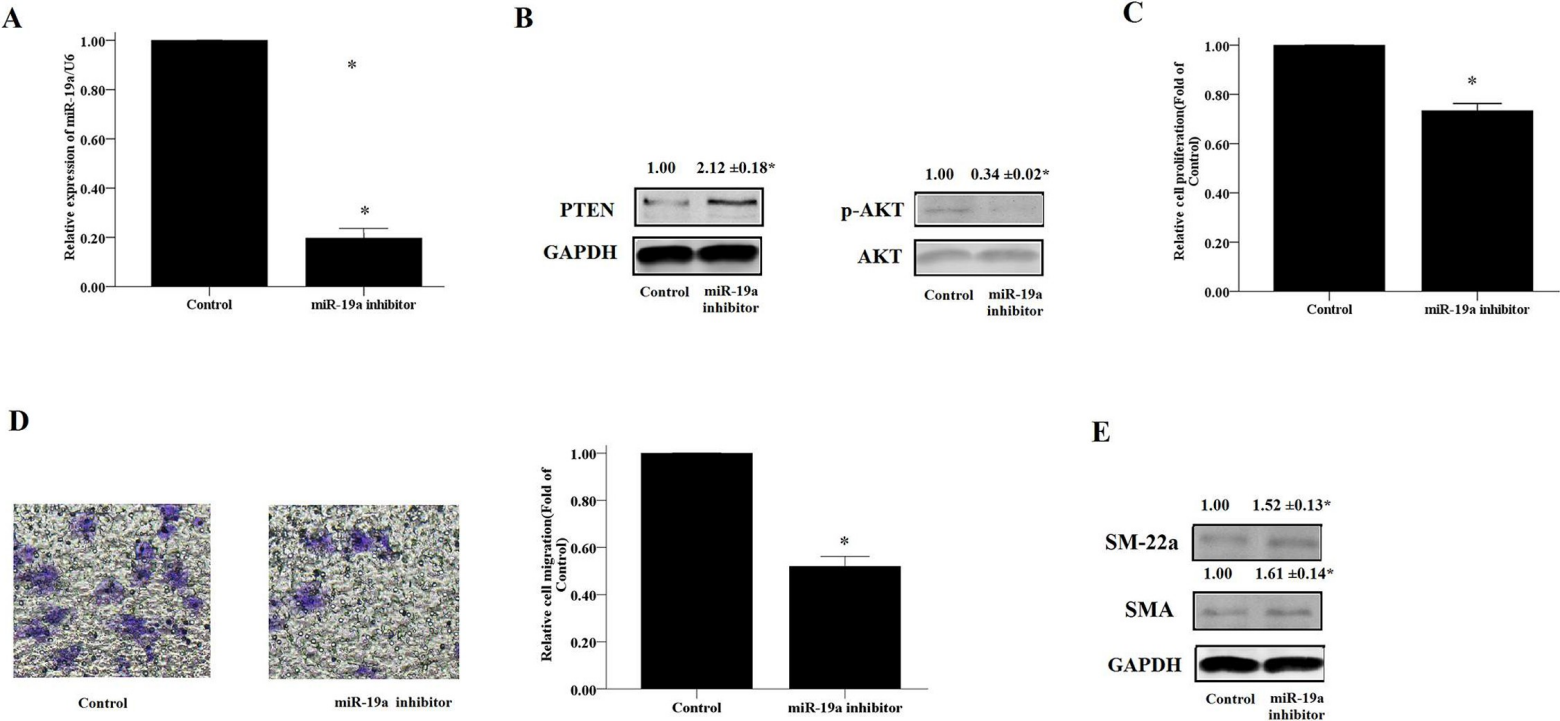
The miR-19a inhibitor suppresses the proliferation and migration of HASM cells. HASM cells were transfected with 100 nM of miR-19a or negative vector control and cultured for 48 h. The transfection efficiency of miRNA-19a was measured by Real-time PCR (A) and the protein levels of PTEN, p-AKT(B), SMA and a-SMA (E) were measured by WB (B). (C) The CCK-8 assay was used to evaluate HASM cell proliferation. (D) The transwell method was performed to evaluate HASM cell migration. The data were expressed as mean±SEM from four independent experiments. * P<0.05 vs. Vector Control cells.

To further confirm the role of miR-19a in regulating proliferation, migration and expression of contractile marker proteins in HASM cells, we transfected the cells with miR-19a inhibitor for 48 h. As shown in Fig 5A, transfection of HASM cells with miR-19a inhibitor led to a 80% inhibition of miR-19a expression compared with that noted for the negative control miR inhibitor. Subsequently, we demonstrated that the miR-19a inhibitor significantly suppressed the proliferation, migration and AKT phosphorylation of HASM cells (Fig 5B–5D). In contrast to these observations, the protein levels of PTEN, SM-22a and SMA were increased in HASM cells transfected with miR-19a inhibitor in comparison with those transfected with negative control miR inhibitor. These results further demonstrated that miR-19a could promote cell proliferation and migration of HASM cells by regulating the PTEN/AKT pathway.

### miR-19 was involved in HMGB1-induced proliferation and migration of HASM cells

**Fig 6:**
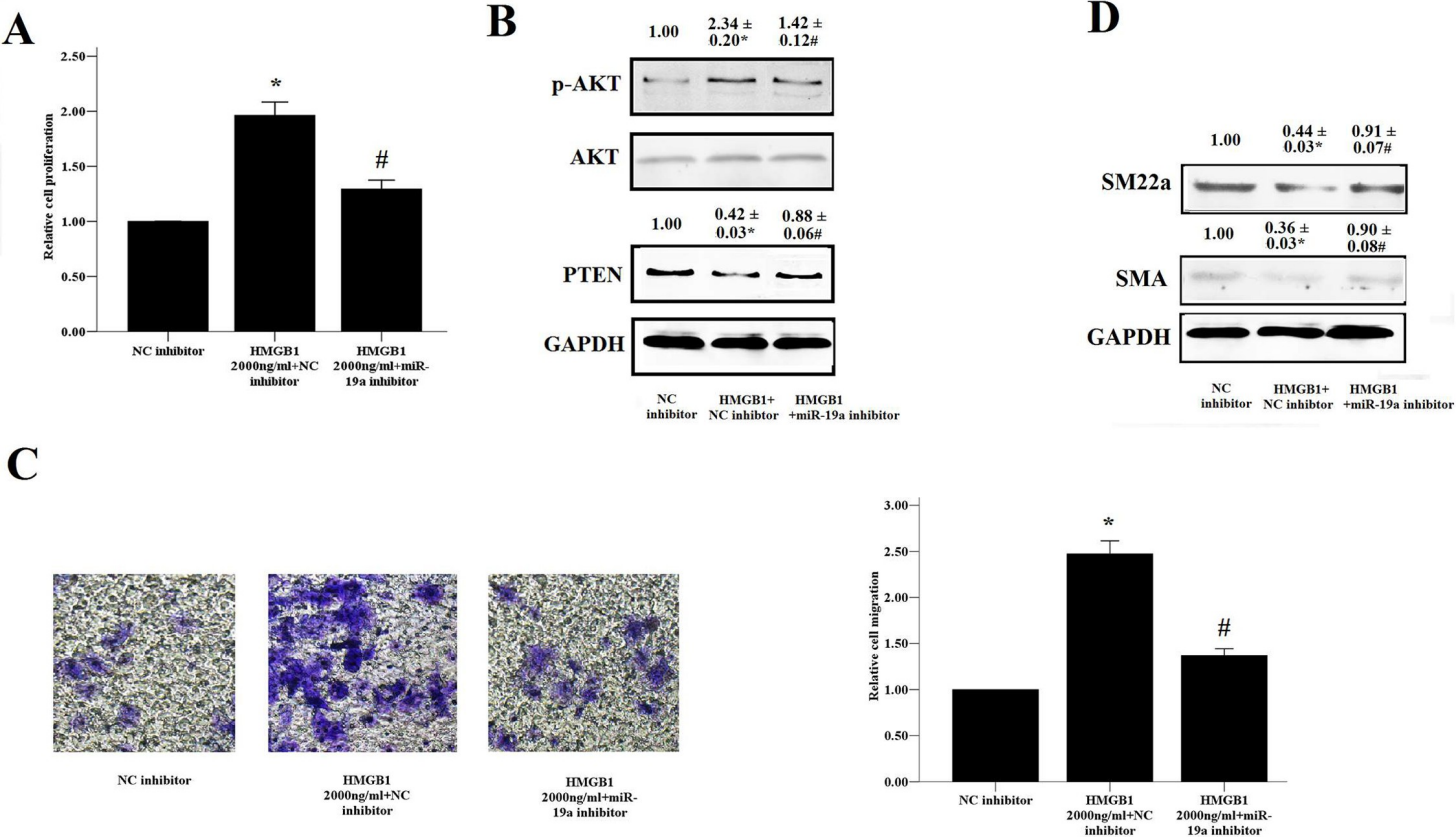
MiR-19 regulates HMGB1-induced proliferation and migration of HASM cells. (A, B) HASM cells were transfected with 100 nM of miR-19a inhibitor in the presence and/or absence of HMGB1 for 48 h and the protein levels of PTEN, p-AKT(B), SMA and a-SMA(D) were detected by WB. The CCK-8 assay was used to evaluate HASM cell proliferation (A) and the transwell method was performed to evaluate HASM cell migration(C). The data were expressed as mean±SEM from four independent experiments. * P<0.05 vs. NC inhibitor cells; #P < 0.05 vs. HMGB1-treated cells (2,000 ng/ml) and NC inhibitor.

To determine whether HMGB1 can promote cell proliferation and migration via the expression of miR-19a, HASM cells were transfected with miR-19 inhibitors and subsequently co-treated with HMGB1 for 48 h. As shown in Fig 6A–6C, HMGB1 (2,000 ng/ml) induced proliferation and migration of HASM cells, whereas the downregulation of miR-19a ameliorated HMGB1-induced cell proliferation, migration and AKT phosphorylation (Fig 6A–6C). Similarly, treatment of the cells with the miR-19a inhibitor significantly reversed the HMGB1-induced decrease of PTEN, SM-22a and SMA protein expression in HASM cells (Fig 6B and 6D). The results indicated that the effects of HMGB1 on HASM cell proliferation and migration were dependent on the alteration in the miR-19a expression levels. In addition, we also found that miR-19 only regulates the proliferation of HASM cells induced by HMGB1,but not PDGF, EGF, TGF-β1(S3 Fig).

### HMGB1 inhibited PTEN via miR-19 targeting of PTEN 3’-UTR

**Fig 7:**
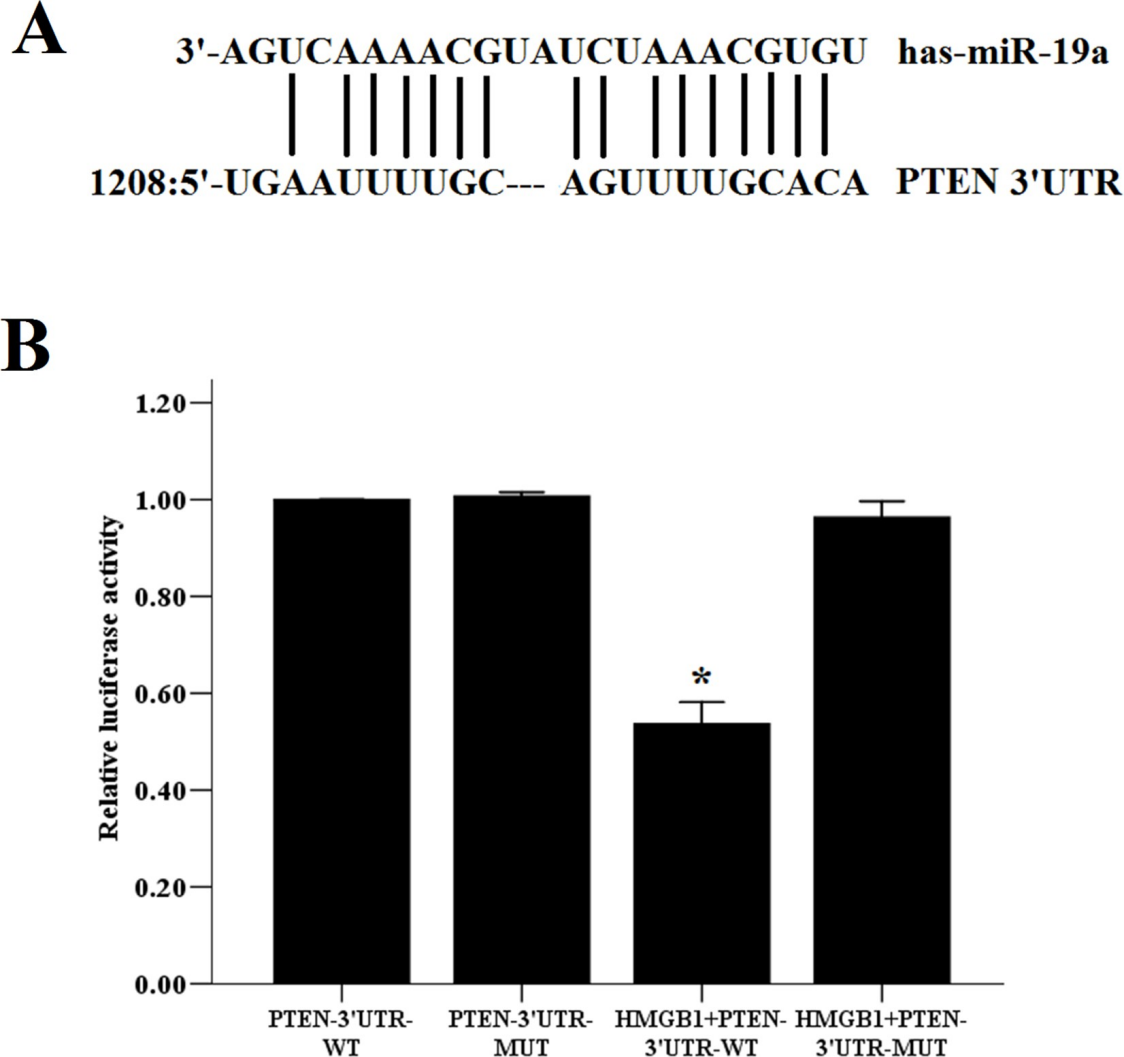
HMGB1 suppresses PTEN by miR-19 targeting the 3-UTR of PTEN. HASM cells were transfected with wild-type or mutant Luc-PTEN-3’UTR dual luciferase reporter plasmids and were subsequently treated with HMGB1 (2,000 ng/ml). The relative luciferase activity was determined by the dual-luciferase reporter assay. The data were expressed as the mean±SEM of four independent experiments. * P<0.05 vs. control group.

Having shown that HMGB1 decreased the expression levels of PTEN, we wanted to investigate whether HMGB1 could regulate PTEN expression via miR-19a. HASM cells were transfected with a PTEN-3′UTR-WT and/or a PTEN-3′UTR-MUT plasmid using lipofectamine 2000 reagent (Invitrogen). Following transfection, HMGB1 (2,000 ng/ml) was added to each well for 48 h. The luciferase activity of PTEN-3′UTR-WT or PTEN-3′UTR-MUT HASM cells treated with HMGB1was evaluated. As shown in Fig 7B, HMGB1 decreased the fluorescence activity by 52.0% in the PTEN-3′UTR-WT group, while in the PTEN-3′UTR-MUT group, this decrease in activity was not detected. The data demonstrated that HMGB1 inhibited PTEN via the upregulation of miR-19 on the target 3’-UTR sequence of PTEN. HMGB1 and/or miR-19a induce proliferation and migration of HASM cells via the PI3K/AKT pathway.

**Fig 8:**
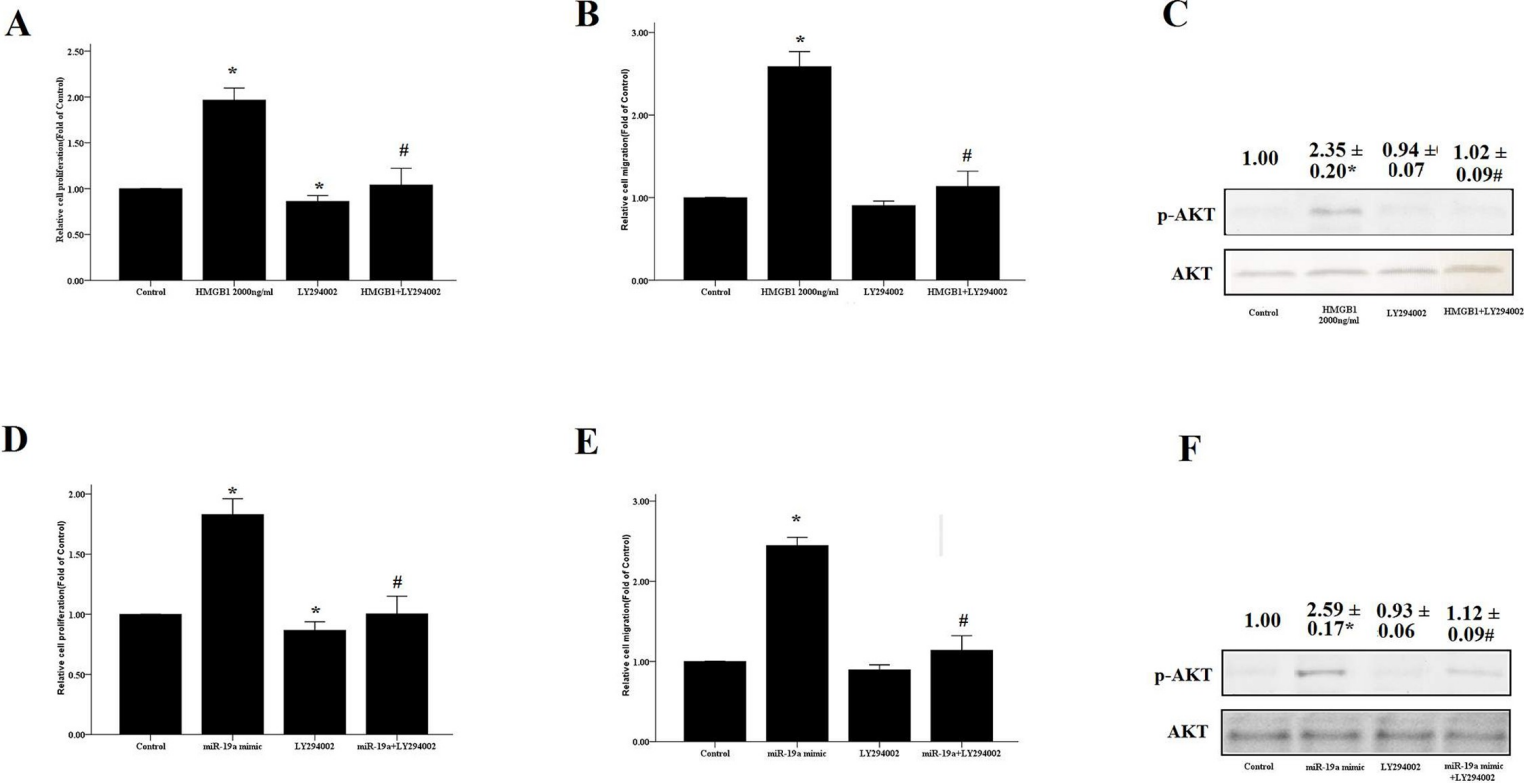
The PI3K/AKT signaling pathway is involved in the HMGB1/miR-19a-induced proliferation and migration of HASM cells. HASM cells were incubated by HMGB1 in the absence or presence of 25 μM of LY294002 and their proliferation and migration were evaluated by the CCK-8 assay (A) and the transwell method (B), respectively. The protein levels of p-AKT and PTEN (C) were detected by WB. HASM cells were transfected with the 50 nM miR-19a mimics or negative vector control and cultured for 48 h. LY294002 (25 μM) was used to inhibit the activation of the PI3K/Akt pathway in the presence of HMGB1 (500 or 2,000 ng/ml), Similarly, the proliferation and migration of HASM cells were evaluated by the CCK-8 (D) and transwell assays (E), respectively and the protein levels of p-AKT (F) were detected by WB; * P<0.05 vs. control group. #P < 0.05 vs. the cells treated with HMGB1 (500 or 2,000 ng/ml) or transfected with the miR-19a mimics.

Using pharmacological inhibitors, we further confirmed the role of the PI3K/AKT pathway in HMGB1- and/or miR-19a-induced proliferation and migration of HASM cells. We found that HMGB1 significantly increased the proliferation and migration of HASM cells and resulted in elevated p-Akt levels, although these processed were inhibited by co-treatment of the cells with a PI3K/AKT inhibitor LY294002 (Fig 8A–8C). Similarly, LY294002 further inhibited cell proliferation and migration mediated by miR-19a overexpression (Fig 8D–8F). These results indicated that the effects of HMGB1 or miR-19a on the proliferation and migration of HASM cells were dependent on the activation of the PI3K/AKT signaling pathway.

## Discussion

To the best of our knowledge, this is the first report showing that exogenous treatment of HASM cells with HMGB1 can induce their proliferation and migration in a dose-dependent manner. Furthermore, we identified miR-19a as a novel regulator that controls HASM cell proliferation and migration by modulating the PTEN/AKT pathway. More importantly, our report further confirmed that HMGB1 plays a vital role in airway remodeling affecting directly the function of airway smooth muscle cells.

Asthma is characterized by chronic airway inflammation and airway remodeling [1–2], which are accompanied by abnormal airway smooth muscle (ASM) mass. Increased airway smooth muscle mass is present in fatal asthma [15]. The excessive proliferation and migration of HASM cells can directly lead to the production of airway smooth muscle mass [15,20]. HMGB1 has been found to promote the proliferation and migration of various types of tumor cells, such as squamous lung cell carcinoma cells, breast cancer cells and hepatocellular carcinoma cells [21]. In a mouse model, the inhibition of the HMGB1-RAGE interaction can inhibit tumor growth and metastasis by decreasing the activation of p44/p42, p38 and SAP/JNK MAP kinases, which can lead to the expression of matrix metalloproteinase enzymes (MMPs) [22]. A previous study has shown that HMGB1 enhanced proliferation of pulmonary arterial smooth muscle cells (PASMC) by activation of mitogen-activated protein kinases and further activation of the downstream proteins c-Fos and c-Jun, which is in agreement with our findings [17]. Similarly, HMGB1 can also induce *in vitro* proliferation of human PASMCs and enhance their migration in a concentration-dependent manner as determined by an *in vitro* chemotaxis assay [23]. In addition, ASM cells exhibit a contractile phenotype that can switch to a proliferative phenotype following treatment with mitogenic stimuli, such as the platelet-derived growth factor (PDGF) [18]. Proliferative ASM cells are characterized by high proliferative rate and low expression levels of contractile proteins, such as SM-22a and SMA. In the present study, we observed that HMGB1 stimulated the proliferation of HASM cells and further decreased the expression levels of SM-22a and SMA, which suggested that HMGB1 may be a potent and novel stimulus capable of inducing ASM cell phenotypic modulation. Pertinent to this finding, a previous study demonstrated that modulation of the ASM cell phenotype can contribute to the pathogenesis of asthma, notably airway remodeling [18].

MiR-19a is a key member of the miR-17-92 cluster and has been found to be over-expressed in several cancer types. MiR-19a has been implicated in the proliferation and migration of different types of tumor cells, such as gastric cancer cells and lung carcinoma cells [19]. Previous studies have shown that miR-17-92 promotes PASMC proliferation by targeting plasminogen activator inhibitor-1 (PAI-1) [24] and PDZ and LIM domain 5 in a smooth muscle cell-specific miR-17-92 knockout mouse model [25]. To the best of our knowledge, no study has been conducted on the contribution of miRNAs in HMGB1-induced proliferation and migration of HASM cells. Recently, the role of miR-19a in the pathogenesis of allergic asthma has received considerable attention. The expression levels of miR-19a were observed in human airway-infiltrating T cells from asthmatic subjects [11]. Moreover, miR-19a modulated TH2 cytokine production and promoted the inflammatory response by direct targeting of PTEN [11], whereas this miRNA was shown to regulate type 2 innate lymphoid cell homeostasis and function including cytokine expression, by inhibiting SOCS1 and A20 [26]. MiR-19a also has been reported to enhance proliferation of bronchial epithelial cells in severe asthma via the TGF-β receptor 2 signaling [27]. In accordance with the aforementioned experimental studies, we illustrated that inhibition of miR-19a significantly reduced proliferation and migration of HASM cells. More importantly, we found that HMGB1 increased significantly the expression levels of miR-19a in HASM cells. Moreover, we further demonstrated that miR-19a was required for HMGB1-induced proliferation and migration in HASM cells. To the best of our knowledge, this is the first report showing that the HMGB1/miR-19a axis can modulate the functions of the HASM cells. It is well-known that PDGF, EGF and TGF-β1 can promote ASM cell growth and migration [28], but the results of our research showed that miR-19 wasn’t involved in the proliferation of HASM cells induced by PDGF, EGF or TGF-β1.The results mentioned above indicate that the mechanism by which hmgb1 regulates ASM proliferation is different from other cytokines like PDGF and EGF so on.

PTEN is well known as a crucial tumor-suppressor gene in multiple cancers and its expression is downregulated in several types of tumors [29]. PTEN is an important negative regulator of the PI3K/AKT signaling pathway and acts by dephosphorylation of the phosphatidylinositol triphosphate-3-phosphatase (PIP3) [30]. The PI3K/AKT signaling pathway plays a vital role in the regulation of cell proliferation, differentiation and apoptosis [30]. Recently, PTEN and the PI3K/AKT signaling pathway have attracted considerable attention due to their crucial roles in asthma. PTEN protein expression and PTEN activity have been shown to be decreased in an OVA-induced asthma model in mice, whereas administration of the PI3K inhibitors or exogenous PTEN decreased considerably bronchial inflammation and airway hyperresponsiveness in an OVA-induced asthma model [31]. In accordance with these studies, we showed that both HMGB1 and miR-19a increased AKT phosphorylation in HASM cells with reduced PTEN expression. We also demonstrated that PTEN was identified as a target gene of miR-19a in HASM cells (As shown in S1 Fig), which is supported by various previous reports [11,26,32]. It must also be mentioned that previous studies have shown the contribution of other miRNAs in regulating the PI3K/AKT/PTEN pathway [33,34]. Specifically in human ASM cells, TNF-α-induced CD38 expression is regulated by miR-708 directly binding to 3'UTR and indirectly by regulating PI3K/AKT signaling [33]. These interactions have the potential to control airway inflammation, ASM contractility and proliferation. [33] Whether this process occurs following HMGB1 treatment remains unknown. More importantly, we further found that HMGB1 regulated PTEN expression through miR-19. In addition, PTEN can regulate cell survival, growth, differentiation and metabolism by dephosphorylating AKT [11,26,32]. The present study demonstrated that both HMGB1 and miR-19a could activate the PI3K/Akt pathway, which was involved in HASM cell proliferation and migration.

The present study indicated for the first time that the HMGB1/miR-19 /PTEN/AKT axis played a very important role in promoting the proliferation and migration of HASM cells. These findings could provide a novel perspective on the molecular mechanism by which HMGB1 exerts its protective effects on HASM cells. More importantly, the results of the present study suggested that targeting of HMGB1/miR-19a in HASM cells may provide a novel therapeutic strategy for preventing ASM hyperplasia.

## Supporting information

S1 Fig PTEN is the target of miR-19a in HASM cells. (a) The putative miR-19a binding sites on 3’-UTR of PTEN mRNA was predicted. (b) After transfection with miR-19a mimics for 48 h, the expression of PTEN in HASM cells was measured by western blot (*p < 0.05 vs control group).(c) The luciferase activity in HASM cells was measured using Dual-Luciferase Reporter Assay System according to the manufacturer’s instruction (*p < 0.05 vs mimic control group).
(TIF)

S2 Fig HMGB1 induces the expression of miR-19a. Primary HASM cells were treated with HMGB12000ng/ml,PDGF 2ng/ml,TGF-B1 10ng/ml and EGF 3nM for 48 h. U6 snRNA was used as an internal control. The data were expressed as mean±SEM from four independent experiments. *P<0.05 vs. control cells.
(TIF)

S3 Fig miR-19 only regulates HMGB1-induced proliferation of HASM cells. HASM cells were transfected with 100 nM of miR-19a inhibitor in the presence and/or absence of HMGB12000ng/ml,PDGF 2ng/ml,TGF-B1 10ng/ml and EGF 3nM for 48h seperately. The CCK-8 assay was used to evaluate HASM cell proliferation. The data were expressed as mean±SEM from four independent experiments. # P<0.05 vs. NC inhibitor cells; *P < 0.05 vs. HMGB1-treated cells 2,000 ng/ml and NC inhibitor.
(TIF)
