# Supplementary figures and images for: miR-19 targets PTEN and mediates high mobility group protein B1(HMGB1)-induced proliferation and migration of human airway smooth muscle cells
Source: PLoS One. 2019 Jun 27;14(6):e0219081. doi: 10.1371/journal.pone.0219081 (PMC6597099; doi:10.1371/journal.pone.0219081)

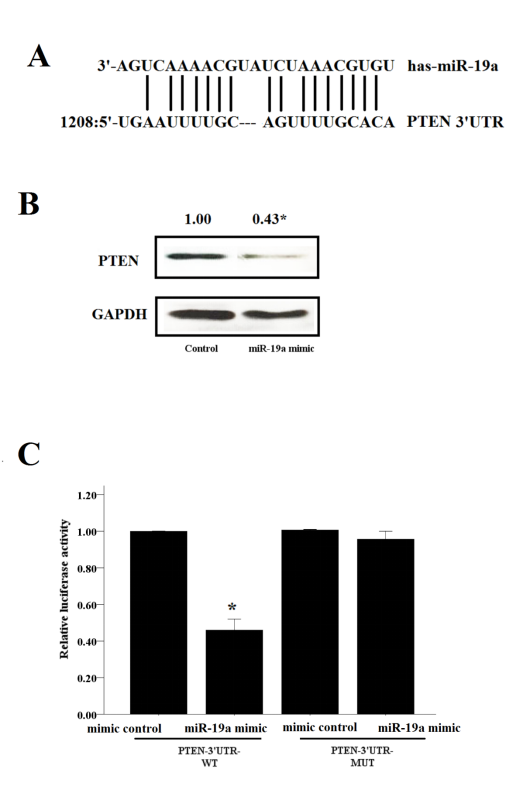

Supplement: S1 Fig — (a) The putative miR-19a binding sites on 3’-UTR of PTEN mRNA was predicted. (b) After transfection with miR-19a mimics for 48 h, the expression of PTEN in HASM cells was measured by western blot (*p < 0.05 vs control group).(c) The luciferase activity in HASM cells was measured using Dual-Luciferase Reporter Assay System according to the manufacturer’s instruction (*p < 0.05 vs mimic control group). (TIF) [file pone.0219081.s001.tif]

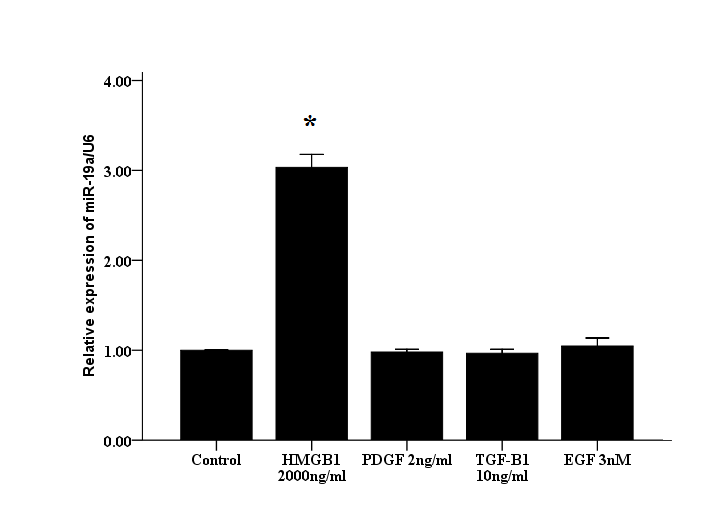

Supplement: S2 Fig — Primary HASM cells were treated with HMGB12000ng/ml,PDGF 2ng/ml,TGF-B1 10ng/ml and EGF 3nM for 48 h. U6 snRNA was used as an internal control. The data were expressed as mean±SEM from four independent experiments. *P<0.05 vs. control cells. (TIF) [file pone.0219081.s002.tif]

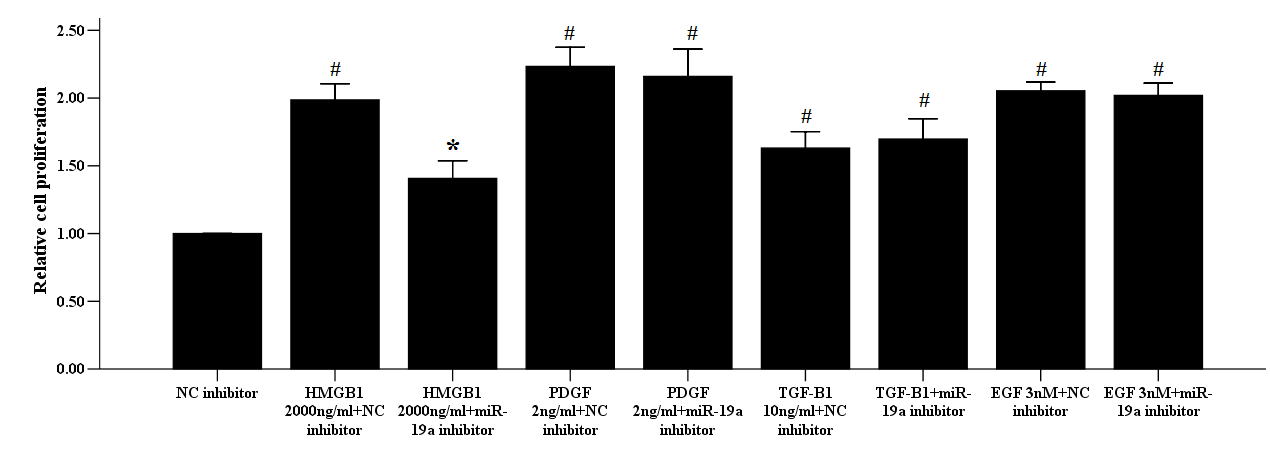

Supplement: S3 Fig — HASM cells were transfected with 100 nM of miR-19a inhibitor in the presence and/or absence of HMGB12000ng/ml,PDGF 2ng/ml,TGF-B1 10ng/ml and EGF 3nM for 48h seperately. The CCK-8 assay was used to evaluate HASM cell proliferation. The data were expressed as mean±SEM from four independent experiments. # P<0.05 vs. NC inhibitor cells; *P < 0.05 vs. HMGB1-treated cells 2,000 ng/ml and NC inhibitor. (TIF) [file pone.0219081.s003.tif]
